# Roxadustat Attenuates the Disruption of Epithelial Tight Junction in Caco2 Cells and a Rat Model of CKD Through MicroRNA-223

**DOI:** 10.3389/fmed.2022.850966

**Published:** 2022-04-13

**Authors:** Ning Qu, Lei Chen, Shanshan Liang, Meng Wei, Lingshuang Sun, Quan He, Jinhong Xue, Meng Wang, Kehui Shi, Hongli Jiang, Hua Liu

**Affiliations:** ^1^Dialysis Department of Nephrology Hospital, The First Affiliated Hospital of Xi‘an Jiaotong University, Xi'an, China; ^2^Department of Blood Transfusion, The First Affiliated Hospital of Xi'an Jiaotong University, Xi'an, China

**Keywords:** chronic kidney disease (CKD), roxadustat, microRNA-223, tight junction proteins (TJPs), hypoxia inducible factor-1α

## Abstract

**Introduction:**

Increasing evidence supports the idea that the disruption of epithelial tight junction proteins (TJPs) caused by accumulation of uremia toxins, such as homocysteine (Hcy), is one of the most important mechanisms underlying the damage of intestinal barrier function (IBF) in chronic kidney disease (CKD). Since the decrease of hypoxia inducible factor-1α (HIF-1α) is reported to be involved in Hcy-induced cell injury, and the upregulation of microRNA-223 (miR-223) plays a vital protective role in the impairment of IBF in the experimental colitis, we investigated the effect of HIF-1α stabilizer roxadustat on the disruption of TJPs induced by Hcy and CKD and the underlying mechanism.

**Methods:**

Chronic kidney disease was induced in rats *via* 5/6 nephrectomy. In a series of experiments, the rats were treated orally with roxadustat of different doses. The expression of tight junction proteins, HIF-1α, and miR-223 was analyzed in different groups by western blotting analysis, RT-qPCR techniques and immunofluorescence. A series of experiments with cultured Caco2 cells was performed.

**Results:**

The results showed that the expression of TJPs (occludin, claudin-1, and ZO-1) decreased significantly, accompanied by the reduction of HIF-1α and miR-223 in Hcy-treated Caco2 cells and colonic mucosa of uremic rats. The reduction of HIF-1α and miR-223 was reversed by roxadustat and the decrease of TJPs expression was attenuated in both Caco2 cells induced by Hcy and colon tissue of CKD rats. Furthermore, transfection with miR-223 mimics increased the expression of TJPs, while transfection with miR-223 inhibitor decreased their expression in Caco2 cells. MiR-223 inhibitor applied before roxadustat treatment partly diminished the effect of roxadustat on TJPs expression in Caco2 cells.

**Conclusion:**

These results indicated that roxadustat attenuated the disruption of epithelial TJPs induced by Hcy in Caco2 cells and the damage of colonic epithelium in CKD rats through the upregulation of miR-223 induced by HIF-1α. A novel insight into the IBF dysfunction in CKD was provided, and it suggests a potential therapeutic use of roxadustat for the IBF dysfunction besides anemia in CKD.

## Introduction

Recently, accumulating evidence has supported the proposal that intestinal barrier function (IBF) is impaired in chronic kidney disease (CKD), especially in end-stage renal disease (ESRD) (1–3). One of the most important underlying mechanisms is that uremic toxins, which accumulate as the renal function declines, disturb the expression of tight junction proteins (TJPs) of intestinal epithelial cell, such as claudin-1, occludin, and zonula occludens (ZO)-1 (4). Since TJPs are crucial for keeping intestinal permeability, the loss of TJPs will lead to more toxins derived from gut penetrating into blood, which, in turn, aggravates the injury of IBF. This vicious cycle between uremic toxins and impaired IBF subsequently results in the progression of CKD and its numerous complications (5). As one of the most important uremia toxins, homocysteine (Hcy) with an elevated level in all forms of CKD (6) has been demonstrated to be the main culprit for the TJPs loss and the IBF impairment in our previous study (7). Therefore, the strategies aimed at protecting individuals with CKD from intestinal barrier injury are urgently in need.

Despite the well-documented mechanisms underlying the pathogenicity of Hcy, such as oxidative stress (8), endoplasmic reticulum stress (9), autophagy (10), recent study has demonstrated that the hypoxia inducible factor-1α (HIF-1α) signal pathway was also involved in the Hcy-induced cell injury (11). The level of HIF-1α significantly decreased when cells are exposed to hyperhomocysteinemia (HHcy). And other studies have revealed that disruption of epithelial HIF-1α resulted in decrease of TJPs and increased epithelial permeability in colitis and eosinophilic esophagitis (12, 13). As such, preventing the decrease of HIF-1α may be a potential way to maintain epithelial barrier function. To our knowledge, the HIF-1α stabilizer is such a kind of a chemical agent that could protect the decrease of HIF-1α effectively. The most advanced HIF-1α stabilizer, roxadustat (also known as FG-4592), is well investigated for the treatment of anemia by targeting HIF-1α with promising results in CKD (14, 15). However, whether roxadustat has protective effects on maintaining the IBF in CKD remains unknown.

MicroRNAs (miRNAs) are a class of approximately 22-nt noncoding RNAs, which negatively regulate gene expression at the posttranscriptional level in a broad array of cell processes. Nowadays, accumulating miRNAs have been recognized to be regulated by HIF-1α (16, 17). Among them, microRNA-223 (miR-223) is found to be upregulated by HIF-1α, while the HIF-1α stabilizer could significantly increase its expression (18). Interestingly, upregulated miR-223 has also been reported to play a vital protective role in the experimental colitis (19), which displayed similar impairment of IBF as occurred in CKD. Thus, we wonder whether HIF-1α stabilizer roxadustat can improve the IBF through increasing the expression of HIF-1α and miR-223 in CKD.

In the present study, we hypothesized that HIF-1α was dysregulated in disruption of TJPs, which contributed to the IBF impairment in CKD. The potential protective effects of roxadustat on TJPs was evaluated, and the involvement of miR-223 in this process was validated *in vitro* and *in vivo* models. These findings might provide new therapeutic strategies for IBF dysfunction of patients with CKD.

## Materials and Methods

### Ethical Statement

This study was conducted in accordance with the criteria outlined in the Guide for the Care and Use of Laboratory Animals. All protocols were approved by the Experimental Animal Ethics Committee, School of Medicine, Xi'an Jiaotong University (XJTULAC2019-1274).

### Experiment Animals

A total of 20 adult male Sprague-Dawley rats (180–220 g) purchased from the Animal Center of the School of Medicine, Xi'an Jiaotong University, Xi'an Shaanxi, China were used for the experiments and were randomly divided into the sham-surgery group (Group SH, *n* = 10) and the uremia group (Group UR, *n* = 10). The animals were housed at room temperature with a 12-h-light/dark cycle, with free access to food and water. The animals were anesthetized with an intraperitoneal injection of 30 mg/kg of 1.5% pentobarbital sodium (Chemical Reagent Factory, Xi'an, China). The rats in the Group UR underwent excision of approximately two-thirds of the left renal tissue during the first laparotomy, and, 7 days later, the whole right kidney was removed during the second laparotomy (i.e., 5/6 nephrectomy was performed). The rats in Group SH underwent two laparotomy procedures without tissue removal or other interventions. On the 20th week after surgery, the animals were sacrificed and the blood samples obtained from the abdominal aorta were centrifuged at 3,000 rpm for 15 min to assess creatinine and urea nitrogen. Renal cortical tissues and colon tissues were used for pathological staining. The remaining samples were stored at −80°C for other investigations.

### Histopathological Examination

The specimens of kidney and colon tissue stored in 4% neutral buffered formalin were embedded in paraffin after routine histological processing. Then, these paraffin-embedded specimens were cut into sections at thickness of 5 μm. After routinely dewaxing and hydration, the sections were dyed with haematoxylin and eosin (H&E, Servicebio, Wuhan, China) and Masson's trichrome staining (Servicebio, Wuhan, China). Histology was performed at magnification of ×400, and pathology and morphological analyses were independently performed by an experienced pathologist blinded to the protocol.

### Cell Culture

Caco2 cells (Shanghai Suer Biotechnology Co. Ltd., Shanghai, China) were cultured in a Dulbecco's modified eagle medium (DMEM) (Life Technologies Corporation, California, USA), supplied with 10% fetal bovine serum (Gibco Company, Gaitherburg, MD, USA) and 1% penicillin-streptomycin (Gibco Company, Gaitherburg, MD, USA) in a humidified incubator with 5% CO_2_ at 37°C. The medium was replaced every 2 days. When cell confluence reached 80%, the cells were treated with 0.25% trypsin (Gibco Company, Gaitherburg, MD, USA) and further passaged. The cells in the logarithmic phase were used for experiment.

### Treatment of Caco2 Cells With Hcy and HIF-1α Stabilizer (Roxadustat)

Caco2 cells in the logarithmic phase were inoculated in 6-well plates at intensity of 2 × 10^5^ cells/well and cultured overnight in a 5% CO_2_ incubator at 37°C. The cells were divided into four groups according to different treatments (3 wells for each group) for 48 h: The roxadustat group treated with 50 μmol/L roxadustat (IR0420, Solarbio), the Hcy group treated with 0.5 mmol/L Hcy (H4628, Sigma), roxadustat plus the Hcy group treated with both roxadustat and Hcy, and the control group without any treatment.

### Cell Transfection

Cells in the exponential phase of growth were plated in six-well plates at 2 × 10^5^ cells/well and cultured for 24 h (3 wells for each group). Synthetic miR-223 mimics/inhibitor and negative control (NC) (Genepharma Technology Co. LTD, Shanghai, China) were transfected into cells at concentration of 50 nmol/L using Lipofectamine® 2000 (Invitrogen, CA, USA) for 24 h, according to the manufacturer's instructions. After that, the cells were collected for the next test.

To confirm whether miR-223 is involved in the protective effect of roxadustat against the reduction of TJPs induced by Hcy, the miR-223 inhibitor and NC were transfected into Hcy-treated cells for 24 h before roxadustat treatment. The transfected cells were cultured in a medium with or without 50 μmol/L roxadustat for 24 h, respectively. The cells were harvested at 48 h post-transfection for further analysis. All the experiments were repeated three times.

### Animal Vivo Validation

Another 40 adult male Sprague-Dawley rats (180–220 g) were purchased from the Animal Center of the School of Medicine, Xi'an Jiaotong University. Roxadustat was purchased from the First Affiliated Hospital of Xi'an Jiaotong University. All the rats were randomly divided into the sham-surgery group (Sham, *n* = 10) and the uremia group (*n* = 30). A rat model with CKD was established as previously reported.

On the 20th week after surgery, renal function was evaluated by blood sampling from the rat inner canthus vein. Serum creatinine and urea nitrogen levels were determined to confirm the molding succeeded. Then, the uremia group rats were randomly allocated into 3 groups: the control group (CKD, *n* = 10); the low-dose group (CKD-LOW, *n* = 10, 7.5-mg/kg roxadustat); the high-dose group (CKD-HIGH, *n* = 10, 10.-mg/kg roxadustat). Roxadustat was administered by oral gavage, TIW (Monday, Wednesday, and Friday) for 6 weeks (the dosing phase). The doses were based on the most recently recorded body weight. Six weeks later, the animals were sacrificed. The blood samples and colon tissues were taken out and then treated as before.

### Reverse Transcription-Quantitative Polymerase Chain Reaction (RT-qPCR)

According to the manufacturer's manual, the total RNA was extracted from tissue or cells using an Ultrapure RNA Kit (CWbio, Beijing, China), and the complementary DNA (cDNA) was synthesized with the PrimerScript real-time reagent kit (TakaraBio Technology, Dalian, China). RT-qPCR was performed using the Fast Start Universal SYBR Green Master (Roche, Basel, Switzerland) on the real-time PCR system (SLAN, Shanghai, China). All mRNA expression levels were normalized to GAPDH. The U6 small nuclear RNA was used as internal control for miRNA detection. All reactions were performed in triplicate. The ΔΔCt method was used for relative quantification. All PCR primer sequences were designed and synthesized by Samgon Biotechnology Company (Shanghai, China). The primer sequences are shown in Table 1.

**Table 1:** Related genes and internal reference-specific primers.

**Organism**	**Gene**	**Primes types**	**Primers sequences**	**Sequence accession number**
**Human**	**Has-miR-223-3p**	Loop	GTCGTATCCAGTGCAGGGTCCGAGGTATTCGCACTGGATACGACTGGGGT	–
		Forward	GCGCGTGTCAGTTTGTCAAAT	–
		Reverse	AGTGCAGGGTCCGAGGTATT	–
	**U6**	Forward	CGCTTCGGCAGCACATATAC	6-25from NR_104084
		Reverse	AAATATGGAACGCTTCACGA	105- 86from NR_104084
	**ZO-1**	Forward	CTAAGGGAGCACATGGTGAAGGTAA	4792-4816 from NM_001330239
		Reverse	GTCGGGCAGAACTTGTATATGGTTT	5052-5028from NM_001330239
	**Occludin**	Forward	AACTTCGCCTGTGGATGACTTCAG	1249- 1272 from NM_NM_002538
		Reverse	TTTGACCTTCCTGCTCTTCCCTTTG	1353-1329 from NM_NM_002538
	**Claudin-1**	Forward	GAAGATGAGGATGGCTGTCATTGGG	582-606 from NM_021101
		Reverse	GGTAAGAGGTTGTTTTTCGGGGAC	820-797 from NM_021101
	**GAPDH**	Forward	TCAAGAAGGTGGTGAAGCAGG	795-815 from NM_001357943
		Reverse	TCAAAGGTGGAGGAGTGGGT	909-890 from NM_001357943
**Rats**	**Rno-miR-223-3p**	Loop	GTCGTATCCAGTGCAGGGTCCGAGGTATTCGCACTGGATACGACTGGGGT	–
		Forward	GCGCGTGTCAGTTTGTCAAAT	–
		Reverse	AGTGCAGGGTCCGAGGTATT	–

### Immunofluorescence

For immunofluorescence staining, 4-μm paraffin sections were prepared, and, after dewaxing and hydration, the samples were incubated in 3% H_2_O_2_ for 15 min at room temperature. After three washes in PBS for 5 min each, the samples were incubated in 5% BSA at room temperature for 30 min. The first antibody of ZO-1 (GB111402, Servicebio) and occludin (GB111401, Servicebio) was incubated with the sections overnight at 4°C. The second fluorescent antibody was incubated with the sections at room temperature for 50 min. After rinsing with PBS and then staining with DAPI, the paraffin sections were screened by a scanner (Pannoramic DESK, P-MIDI, P250, 3D HISTECH). DAPI-stained nucleus was in blue; positive occludin protein expression was in red; and positive ZO-1 protein was in green.

### Western Blotting

Total proteins were extracted and solubilized in a RIPA buffer containing proteinase inhibitors (1% cocktail and 1 mmol/L phenylmethylsulfonyl fluoride, Sigma-Aldrich, USA). After the determination of protein concentration with a BSA assay kit (Pierce, Rockford IL, USA), 40 μg of total protein from each sample was separated by 10% SDS-PAGE at 70 V for 30 min, followed by 110 V until the end and transferred to PVDF membranes (Millpore, USA). The membranes were incubated with the primary antibodies overnight at 4°C (Abcam Technology, UK: HIF-1α, 1:2,000; claudin-1, 1:4,000; occludin, 1:1,500; GAPDH, 1:2,000; Thermo Fisher Scientific, USA: ZO-1, 1:1,000), followed by incubation with the peroxidase-conjugated secondary antibodies (Boster Biological Technology, Wuhan, China, 1:5,000) at room temperature for 1 h. The protein bands were detected with the enhanced chemiluminescence (ECL) detection system (Pierce, Rockford IL, USA) and quantitated by densitometry using Image J Software.

### Statistical Analysis

All the experiments were performed in triplicate, and quantitative data were described with mean ± standard deviation (SD). The statistical analyses were performed using SPSS 26.0 software (SPSS, Inc., Chicago, IL, USA), and unpaired Student *T*-test was conducted for comparison between two groups. A *p*-value of < 0.05 (*p* < 0.05) was considered to be statistically significant.

## Results

### The Expression of Tight Junction Proteins (TJPs) Decreased Accompanied by Reduction of HIF-1α and miR-223 in the Colonic Mucosa of Uremic Rats

**Figure 1:**
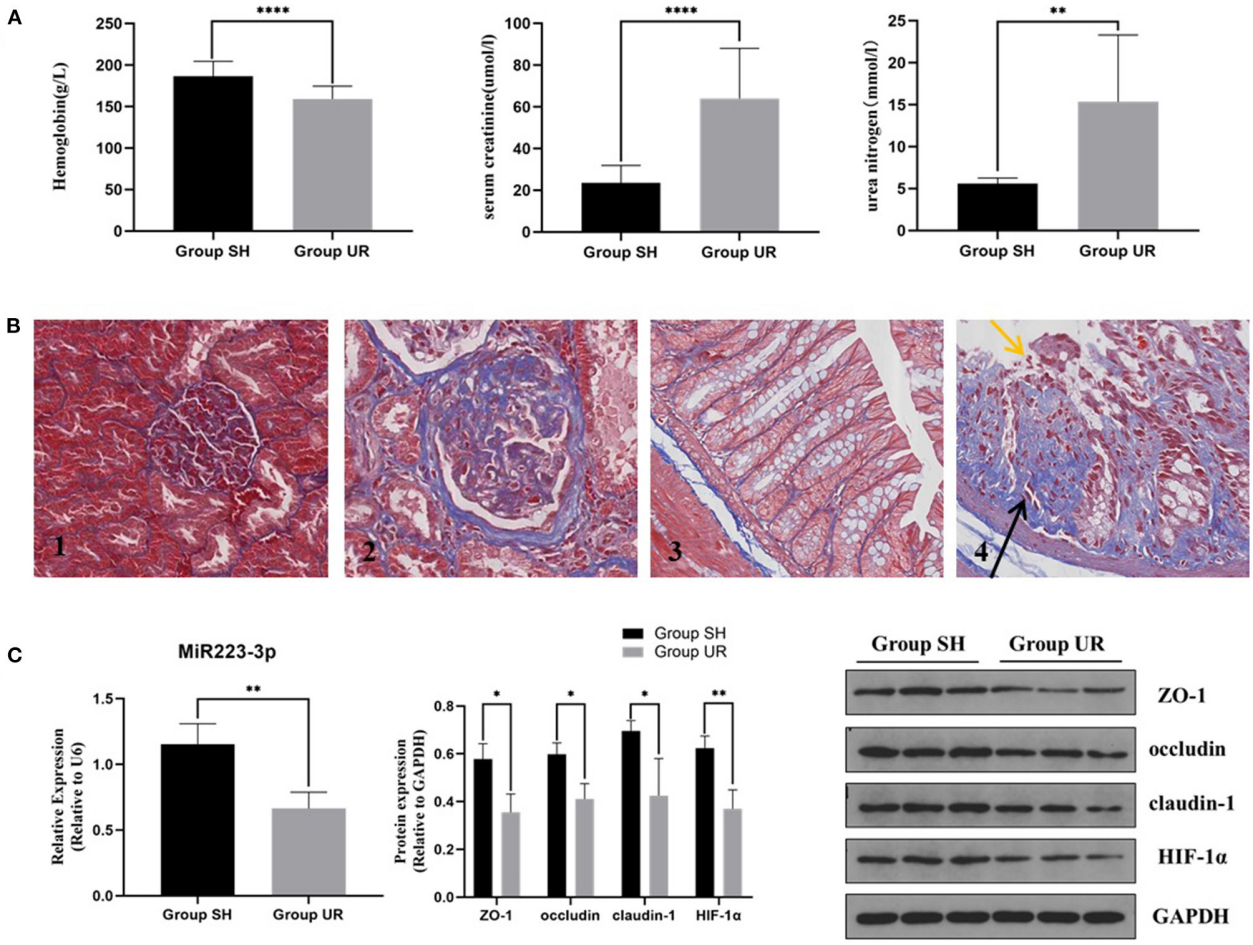
The comparison between the sham-surgery group (Group SH) rats and the uremia group (Group UR) rats **(A)** Hemoglobin, serum creatinine, and urea nitrogen of the rats in Group SH and Group UR. **(B)** Masson's trichrome staining of renal tissue (magnification × 200) **(B1,B2)** and Masson's trichrome staining of colon tissue (magnification × 400) **(B3,B4)**; yellow arrows indicate loss of epithelial cells and distortion of mucosa. Black arrows indicate collagen deposition. **(C)** MiR-223 relative expression and protein expressions in Group SH and Group UR (*p < 0.05, **p < 0.01, ****p < 0.0001).

Compared with the sham-surgery group (Group SH), the hemoglobin in the rats of the uremia group (Group UR) was significantly decreased, while the serum creatinine and urea nitrogen levels were significantly increased (shown in Figure 1A). The staining showed that the residual kidney of Group UR appeared with enlargement of the glomerular volume, increase of mesangial matrix, glomerulosclerosis, and interstitial fibrosis. The pathological changes and the impaired renal function levels together indicated that the CKD models were successful. Colonic mucosa in the Group SH rats showed a normal appearance with intact epithelium. In contrast, mucosa in the Group UR rats showed mucosal damage and increased collagen deposition (shown in Figure 1B). Meanwhile, the expression of TJPs, including ZO-1, occludin, and claudin-1, was markedly decreased in the colon tissue of Group UR compared with those of Group SH (shown in Figure 1C). Furthermore, we found the relative expression of miR-223 and HIF-1α protein expression were also decreased in the colon tissue of Group UR when compared to that in Group SH (shown in Figure 1C).

### Roxadustat Reversed the Reduction of HIF-1α, miR-223, and TJPs Expression Induced by Hcy in Caco2 Cells

**Figure 2:**
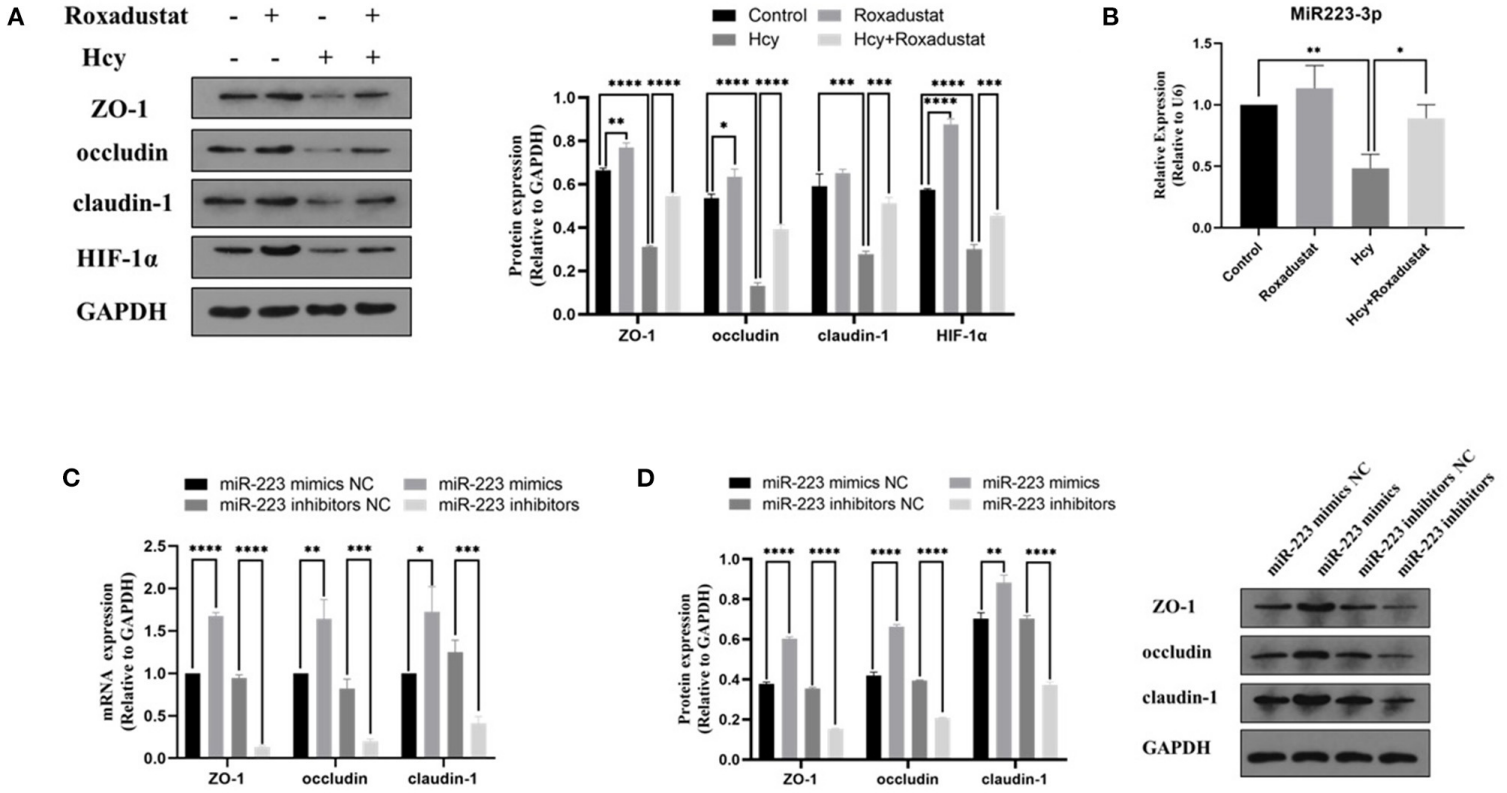
Effect of Hcy and roxadustat on the expression of miR-223, HIF-1α, and TJPs in Caco2 cells. **(A)** Western blotting analysis and quantification of HIF-1α, ZO-1, occludin, and claudin-1. **(B)** Quantitative reverse transcription-polymerase chain reaction (qRT-PCR) detection of miR-223 expression levels. The effect of upregulation or downregulation of miR-223 on the expression of ZO-1, occludin, and claudin-1 in Caco2 cells. **(C)** Quantitative reverse transcription-polymerase chain reaction (qRT-PCR) detection of ZO-1, occludin, and claudin-1. **(D)** Western blotting analysis and quantification of ZO-1, occludin, and claudin-1 (*p < 0.05, **p < 0.01, ***p < 0.001, ****p < 0.0001).

In Caco2 cells, following the stimulation with Hcy (0.5 mmol/L), the protein expression of HIF-1α, ZO-1, occludin, and claudin-1 decreased significantly (shown in Figure 2A). However, administration with roxadustat significantly attenuated the decrease of HIF-1α and TJPs expression in Caco2 cells induced by Hcy (shown in Figure 2A). Compared to the control group, roxadustat significantly increased the expression of HIF-1α, occludin, and ZO-1 (shown in Figure 2A). Meanwhile, the expression of miR-223 dramatically decreased in Hcy-treated Caco2 cells compared with control (shown in Figure 2B), and roxadustat significantly attenuated the decrease expression of miR-223 in Caco2 cells induced by Hcy (shown in Figure 2B).

### The Effect of miR-223-Mimics/Inhibitor on the Expression of TJPs in Caco2 Cells

In order to characterize the effect of miR-223 on the expression of TJPs, the expression levels of ZO-1, occludin, and claudin-1 were detected with RT-qPCR and Western blotting. As shown in Figures 2C,D, transfected Caco2 cells with miR-223 mimics significantly increased the levels of ZO-1, occludin, and claudin-1 at both mRNA and protein levels when compared with the control group (Caco2 cells transfected with miR-223 mimics NC). However, transfection with miR-223 inhibitors significantly decreased the expression levels of ZO-1, occludin, and claudin-1 when compared with the control group (Caco2 cells transfected with miR-223-inhibitor NC). Together, these data suggested that miR-223 promoted the expression of TJPs in Caco2 cells.

### Roxadustat Attenuates the Hcy-Induced Reduction of TJPs Possibly *via* Mediating miR-223

**Figure 3:**
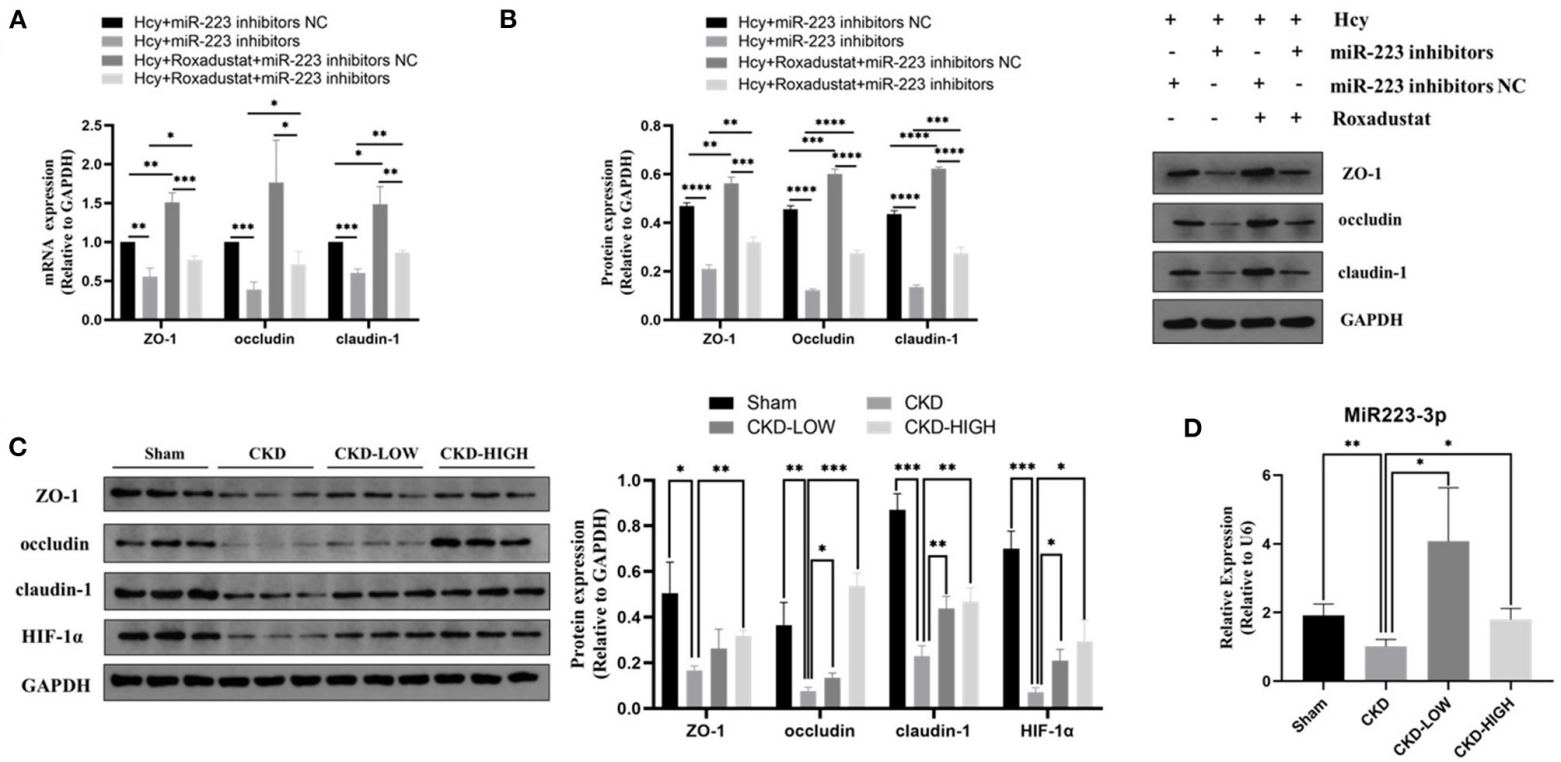
Effects of roxadustat on protein expression of ZO-1, occludin, and claudin-1 were blocked by miR-223-inhibitor in Caco2 cells. **(A)** Quantitative reverse transcription-polymerase chain reaction (qRT-PCR) detection of ZO-1, occludin, and claudin-1. **(B)** Western blotting analysis and quantification of ZO-1, occludin, and claudin-1. Effect of roxadustat on the expression of miR-223 and proteins in colon tissues of CKD rats. **(C)**Western blotting analysis and quantification of ZO-1, occludin, claudin-1, and HIF-1α. **(D)** Quantitative reverse transcription-polymerase chain reaction (qRT-PCR) detection of MiR-223 (*p < 0.05, **p < 0.01, ***p < 0.001, ****p < 0.0001).

To confirm whether miR-223 was involved in the protective effect of roxadustat against the reduction of TJPs induced by Hcy, we transfected the cells with miR-223 inhibitors before roxadustat treatment. As shown in Figures 3A,B, miR-223 inhibitors significantly decreased the expression of TJPs, while roxadustat significantly increased the expression of TJPs at mRNA and the protein level when compared with miR-223 inhibitors NC. The expression levels of TJPs in the group of Hcy + roxadustat + miR-223 inhibitors NC were significantly higher than that in the group of Hcy + roxadustat + miR-223 inhibitors at both mRNA and the protein level. These results indicated that roxadustat attenuates the Hcy-induced reduction of TJPs, at least partially, *via* mediating miR-223.

### Roxadustat Reversed the Reduction of TJPs Protein Expression in Colon Tissues of Uremic Rats Possibly by Increasing miR-223 Expression

The 5/6-nephrectomy rat model was used to verify the effect of roxadustat *in vivo*. We detected the role of roxadustat in HIF-1α and TJPs, including ZO-1, occludin, and claudin-1, by immunoblotting and found that roxadustat reversed the downregulation of ZO-1, occludin, claudin-1, and HIF-1α in response to CKD. The high-dose roxadustat significantly increased the expression levels of ZO-1, occludin, claudin-1, and HIF-1α (shown in Figure 3C), while the low-dose roxadustat only increased the expression levels of occludin, claudin-1, and HIF-1α significantly (shown in Figure 3C) compared with the CKD group. Furthermore, the relative expressions of miR-223 were also increased significantly in the colon tissue of CKD-LOW and CKD-HIGH groups when compared to that in the CKD group (Figure 3D).

**Figure 4:**
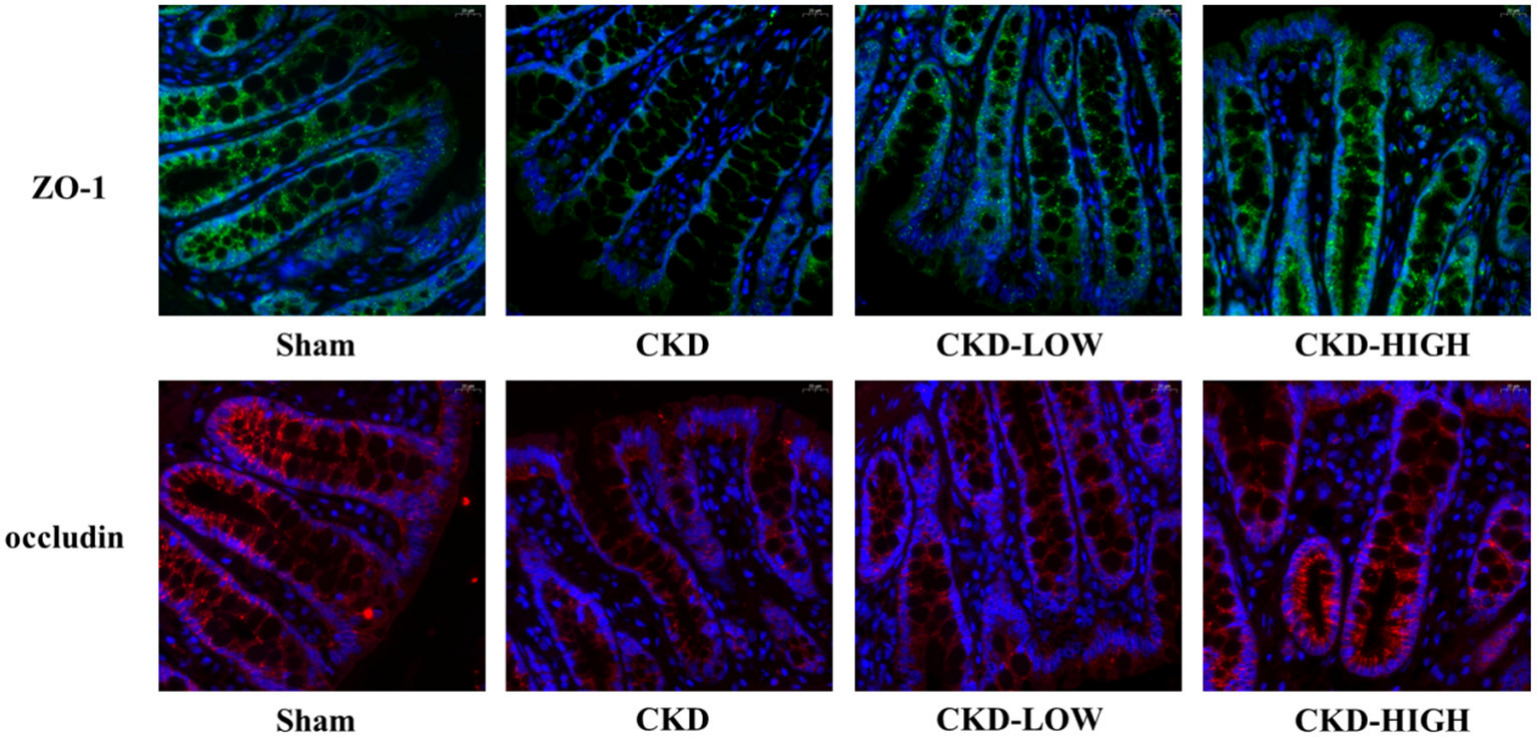
Representative photomicrographs of ZO-1 immunofluorescence (green) and occludin immunofluorescence (red) in the colon of rats. Colon tissue from the Sham group rats exhibited continuous, intense ZO-1 and occludin immunofluorescence in the epithelial cells; whereas colons from the CKD group rats exhibited light, discontinuous ZO-1 and occludin immunofluorescence. The CKD-LOW group rats had continuous ZO-1 and occludin immunofluorescence. The CKD-HIGH group rats displayed the fewer amounts of ZO-1 and occludin immunofluorescence than the Sham group but more than the CKD-LOW group.

To determine if there is an overall loss of tight junctions or a change in the distribution of tight junction protein expression, we used immunofluorescence to qualify the staining patterns of ZO-1 and occludin (Figure 4). The integrity of the intestinal barrier in Sham group rats appeared to be intact, as indicated by the continuous and intense ZO-1 and occludin immunofluorescence. In the CKD group, the immunofluorescence signals of ZO-1 and occludin were disrupted and decreased. After roxadustat treatment, the protein expression intensity of ZO-1 and occludin in CKD-LOW and CKD-HIGH groups was remarkably enhanced and higher than the CKD group. Moreover, the increase of ZO-1 and occludin expression in the CKD-HIGH group was more obvious than that in the CKD-LOW group.

## Discussion/Conclusion

During the past decade, the impairment of IBF in CKD has attracted extensive attention of nephrologists. One of the main features of the IBF impairment in CKD was a large reduction of TJPs, resulting in an increase of intestinal permeability. Vaziri et al. firstly observed the significant decrease of TJPs in the colonic mucosa *in vivo* study with uremic rats (2). Then, other studies obtained similar results in an *in vivo* study with the patients with CKD (3) and an *in vitro* study with human colonocytes, which were incubated with the plasma from the patients with CKD (4, 20). Moreover, the impairment of IBF has been reported to contribute to the progression of CKD and its complications (5, 21). Although the crucial role of the IBF impairment in CKD has been widely recognized, the underlying mechanisms are still remained to being explored, even less research has been conducted on the therapeutic strategies for it. In the present study, we confirmed the reduction of TJPs in both CKD animals and Hcy-treated Caco2 cells, which is consistent with previous studies. In addition, we also found a decrease of HIF-1α and miR-223 in CKD rats and Hcy-treated Caco2 cell. Considering that aberrant expression of HIF-1α may be involved in the IBF impairment, we applied HIF-1α stabilizer roxadustat to the Hcy-treated Caco2 cells and 5/6-nephrectomy CKD rats. The results showed that roxadustat could significantly reverse the decrease of TJPs induced by Hcy and CKD.

Accumulating uremic toxins were thought to be a leading cause of disruption of IBF in CKD (4). Hcy, a representative uremia toxin, increases in the early stages of CKD, and achieves 3–5 times higher than normal in ESRD (6). In our previous study, we demonstrated that elevated Hcy significantly increased intestinal permeability and reduced the expression of TJPs, including occludin, claudin-1, and ZO-1, in uremic rats (7). In this study, the decreases of above TJPs were also observed in Hcy-treated Caco2 cell *in vitro* and colon tissue of CKD rats *in vivo*. Occludin and claudin-1 belong to adhesive transcellular proteins, which link the plasma membranes of the adjacent cells to form the barrier against diffusion of fluids and solutes (22). ZO-1, known as actin-binding cytosolic proteins, serves as the anchor and regulates the organization of the apical junction complex (23). The reduction of these TJPs directly leads to the disruption of structural function of tight junction. As such, our findings in this study further confirmed the harmful role of Hcy in the IBF impairment. Moreover, we found expression of HIF-1α also decreased in both colonic mucosa of uremic rats and Hcy-treated Caco2 cells, indicating that aberrant expression of HIF-1α may be involved in the IBF impairment caused by Hcy. The phenomenon that Hcy attenuates the HIF-1α level has been reported in previous studies. Veeranki et al. reported the levels of HIF-1α significantly decreased in skeletal muscles in a murine model for HHcy (24). Fang et al. showed similar decrease of HIF-1α in Hcy-treated hippocampal neurons *in vitro* (11). Although the mechanism of Hcy on the expression of HIF-1α remained unclear, these results, together, suggested that the decrease of HIF-1α was involved in the Hcy-induced injury.

Hypoxia-inducible factor-1 (HIF-1) is a master transcriptional regulator of cellular response to hypoxia. It is a heterodimer composed of an oxygen labile α subunit (HIF-1α) and a constitutively expressed β subunit (HIF-1β) (25). Under normoxic conditions, HIF-1α is rapidly degraded by the ubiquitin-proteasome system. In hypoxia, HIF-1α escapes degradation and translocates into the nucleus, forming HIF-1 heterodimer with HIF-1β and transactivating genes involved in hypoxic adaptation (26). Despite the hypoxia conditions, pharmacological action of HIF-1α stabilizers, which inhibit prolyl hydroxylase domain-containing proteins (PHDs), could also prevent HIF-1α from degradation (27). Since a number of barrier-protective genes are critically regulated by HIF-1, preventing HIF-1α from degradation has been documented to be protective for the IBF impairment in murine experimental colitis (28). Shao et al. also reported the absence of intestinal HIF-1 alpha exacerbates gut leakiness, leading to an increased translocation of bacteria (29). In our study, we applied roxadustat, the most advanced HIF-1α stabilizer, to treat the tight junction disruption in both the Hcy-induced Caco2 cells and CKD rats and found it attenuated the decrease of TJPs. Roxadustat, a novel oral lyprolyl hydroxylase inhibitor (PHI), activates HIF-1α by inhibiting the production of PHD-2 (30) and has been well investigated for treatment of anemia in patients with CKD (31, 32). The newly published studies reported that roxadustat improved anemia and was associated with continued efficacy in phase three clinical trials of Chinese patients with CKD (14, 15). In addition, Yang et al. showed that roxadustat ameliorated cisplatin-induced kidney injury *via* HIF-1α activation (33). These findings indicated the considerable clinical perspectives of roxadustat in patients with kidney disease. Nevertheless, the role of roxadustat in the impairment of IBF in CKD has not been previously reported. Our study firstly validated that roxadustat reversed the decrease of TJPs induced by Hcy and CKD, which potentially extended the clinical use of roxadustat for treating disruption of IBF besides anemia in patients with CKD.

In the present study, a decrease of miR-223 in CKD rats and Hcy-treated Caco2 cells was also found. Somewhat similarly, miR-223 decreased in a murine model of CKD, and this has been recently confirmed in patients with CKD stages 4 and 5 (34). Abnormal expressed miRNAs also devote themselves to pathogenesis of diseases with IBF disruption (35, 36). MiR-223 has been reported to be a novel biomarker in patients with colitis (37), and its upregulation plays a vital protective role in the experimental colitis (19, 38). Colitis displayed similar impairment of IBF as occurred in CKD. The decreased miR-223 in both the Hcy-treated Caco2 cells and CKD rats' colon tissue was upregulated by HIF-1α stabilizer roxadustat in the current study. Previous studies demonstrated HIF-1α functioned as a pivotal regulator of miRNAs in various disorders (16, 17, 39). Meanwhile, Zhu et al. have recently shown that miR-223 was upregulated by the HIF-1α stabilizer and downregulated by the HIF-1α inhibitor in macrophages (18). In the current study, the expression of TJPs was increased by transfection with miR-223 mimics while decreased by transfection with the miR-223 inhibitor in Caco2 cells. Additionally, the effect of roxadustat on the expression of TJPs was partly diminished when the miR-223 expression was inhibited by miR-223-inhibitor before roxadustat treatment in Caco2 cells. These findings indicated that roxadustat attenuated the disruption of epithelial tight junction induced by Hcy *via* mediating microRNA-223, at least partially.

The possible binding sites of miR-223 in ZO-1, occludin, and claudin-1 mRNAs have been predicted, and the results indicated that these mRNAs were not the target genes of miR-223. Therefore, miR-223 was thought to have an indirect effect on tight junction proteins. The miR-223 targets a variety of factors, including TLR4, PI3K/AKT, PARP-1, HDAC2, ITGB3, CXCL2, CCL3, IL-6, IFN-I, STMN1, IL-1β, IL-18, Caspase-1, NF-κB, NLRP3, and so on (40). Among them, the targeted regulatory effect of miR-223 on NLRP3 has been fully studied. The miR-223 suppresses NLRP3 expression by binding to its conserved binding site in the 3′UTR of NLRP3 and thus inhibits NLRP3 inflammasome activation (41, 42). Considering that NLRP3 has been proved to downregulate the expression of ZO-1, occludin, and claudin-1 in previous studies (43–46), the regulation of miR-223 on TJPs may be accomplished through the targeted regulation of NLRP3. However, it still needs to be clearly proved. Further studies might be needed to confirm our hypothesis.

In summary, the present results demonstrated that HIF-1α, TJPs, and miR-223 were decreased in CKD rats and Hcy-treated Caco2 cells, while HIF-1α stabilizer (roxadustat) attenuates the changes. The underlying mechanism may include the upregulation of miR-223 induced by HIF-1α. Our study first clarified the protective effect of the HIF-1α stabilizer (roxadustat, FG-4592) on TJPs and the potential underlying mechanism in uremia toxin-treated Caco2 cells, which may provide a novel insight into the treatment of IBF dysfunction in CKD. As roxadustat is a promising medicine to improve anemia with continued efficacy in patients with CKD, our findings indicated patients with CKD may benefit more from this medicine. Due to the limitation that the protective effect of roxadustat was not validated in clinical trials in our study, further experiments are needed to validate the function of roxadustat in clinical research.

## Data Availability Statement

The original contributions presented in the study are included in the article/supplementary material, further inquiries can be directed to the corresponding author/s.

## Ethics Statement

The animal study was reviewed and approved by the Experimental Animal Ethics Committee, School of Medicine, Xi'an Jiaotong University.

## Author Contributions

NQ and LC did the major works, including cell experiments and animal experiments. SL, MW, LS, QH, JX, and MW helped in animal experiments. KS helped in the statistical analysis. HL and HJ helped in the study design and manuscript writing. All authors contributed to the article and approved the submitted version.

## Funding

This research work was supported by the National Natural Science Foundation of China (Nos. 81870507 and 81900675), the Social Science and Technology Development Project of Shaanxi Province (Grant No. 2019SF-006), the International Science and Technology Cooperation Project of Shaanxi Province (Grant No. 2019KW-32), and the general projects of Shaanxi Science and Technology (Areas of Social Development, No. 2020SF-122).

## Conflict of Interest

The authors declare that the research was conducted in the absence of any commercial or financial relationships that could be construed as a potential conflict of interest.

## Publisher's Note

All claims expressed in this article are solely those of the authors and do not necessarily represent those of their affiliated organizations, or those of the publisher, the editors and the reviewers. Any product that may be evaluated in this article, or claim that may be made by its manufacturer, is not guaranteed or endorsed by the publisher.
